# Effect of Carbon Partition and Precipitation on Wear Resistance of Carburized Layer in Heavy-Duty Gear

**DOI:** 10.3390/ma14226958

**Published:** 2021-11-17

**Authors:** Tianyu Zhang, Jian Wang, Zhizhou Pan, Qing Tao

**Affiliations:** 1 School of Materials and Physics, China University of Mining and Technology, Xuzhou 221116, China; schtyzhang@cumt.edu.cn (T.Z.); wangjian5288@sjtu.edu.cn (J.W.); TS19180022A31@cumt.edu.cn (Z.P.); 2 School of Materials Science and Engineering, Shanghai Jiao Tong University, Shanghai 200240, China

**Keywords:** tempering, carbon partition, precipitation, mechanical property, wear

## Abstract

The carburizing–quenching–tempering process is generally conducted on heavy-duty gear in order to obtain favorable comprehensive mechanical performance. Different mechanical properties could be produced by carbon partition and precipitation. In this study, the carburizing–quenching–tempering process was carried out on low-carbon alloy steel in order to investigate the influence of microstructure evolution and precipitate transition on mechanical behavior and wear resistance under different carburizing/tempering durations. Favorable comprehensive mechanical property and wear resistance could be obtained in favor of long durations of carburizing/tempering. A fatigue-wear model was proposed to describe fatigue crack evolution and damage mechanism on the basis of wear features.

## 1. Introduction

Mechanical properties play a prominent role in the service life of components, which is primarily determined by treatment process and microstructure [1,2]. Surface modification is generally conducted on mechanical components to create favorable comprehensive performance and to optimize their service life [3]. Carburizing–quenching is one of the most commonly used processes for improving surface strength and hardness and wear resistance of heavy-duty gear, which is required to qualify the eminent fatigue life [4,5]. High hardness and strength could be introduced at the surface, and gradient solid solution carbon and microstructure would be brought in from surface to the core through carburizing–quenching, introducing favorable residual compressive stresses [6,7,8]. Nevertheless, the as-quenched carburized layer usually consists of martensite with supersaturated carbon and high dislocation density, which is rich in strength and hardness but is too poor in terms of plasticity and toughness for practical applications [9,10]. It is supposed that heavy-duty gear should possess both high strength and good toughness, i.e., favorable comprehensive mechanical properties to endure long periods of heavy-load cyclic rolling contact [11]. Thus, microstructure evolution induced by subsequent tempering following carburizing–quenching is necessary for improving the toughness through carbon partition and precipitation [12,13,14,15,16], during which different mechanical properties and wear resistance could be generated by diverse carbon partition, precipitates transition and retained austenite decomposition [17,18]. Barrow et al. [19] investigated low-temperature precipitation in quenched–tempered 100Cr6 steel with different tempering durations, during which ε → η → θ carbide evolution and martensite tetragonality transition were characterized to induce distinct mechanical properties. Compared to high carbon steel, Wang et al. [20] achieved better rolling contact fatigue performance in a carburized nanostructured bainitic steel by utilizing the carburizing, tempering and austempering process. In addition, Yang et al. [21,22] prepared a mixed microstructure composed of nanostructured bainite, carbon enriched retained austenite and carbide in the carburized layer of low-carbon alloy steel, which presented eminent wear resistance. Carbon enriched with retained austenite and with high mechanical stability has been observed to be in favor of inhibiting the initiation and propagation of fatigue crack, mainly attributed to carbon partition from the high carbon phase to austenite during preparation [23,24,25,26,27]. Sluggish transformations from the retained austenite with high mechanical stability to martensite during the long period of heavy-load cyclic rolling contact continuously consumed deformation energy and improved strength by introducing beneficial residual stress, making dominant contributions to high-wear resistance [28,29]. In comparison, Suh et al. [30,31] pointed out that during a long period of heavy-load cyclic rolling contact, retained austenite tends to transform into martensite, due to which high density dislocation emerges and piles up around the martensitic interfaces and subsequently results in fatigue cracking and finally resulting in wear spalling. Nevertheless, research on the effect of diverse microstructure evolution and precipitates transition induced by carbon partition during different carburizing/tempering durations on the mechanical property and wear resistance of carburized–quenched–tempered low-carbon alloy steel is still deficient [32].

In this study, carburizing–quenching–tempering process was conducted on low-carbon alloy steel in order to study the effects of diverse microstructure evolution and precipitates’ transition on the mechanical properties and wear resistance of its carburized layer processed with different carburizing/tempering durations. The 18Cr2Ni4WA steel with the composition of Fe-0.16C-1.5Cr-4.25Ni-0.27Si-0.45Mn (wt.%) was vacuum induced melted and used for present work, which could be qualified with favorable comprehensive mechanical property via probable carburizing–quenching–tempering treatment and is generally employed for heavy-duty gears in terms of insufficient toughness for medium/high carbon steel to endure long periods of heavy-duty cyclic rolling contact. On the basis of wear features and wear resistance analysis, a fatigue-wear model was proposed to describe fatigue crack evolutions and damage mechanism.

## 2. Materials and Methods

**Figure 1:**
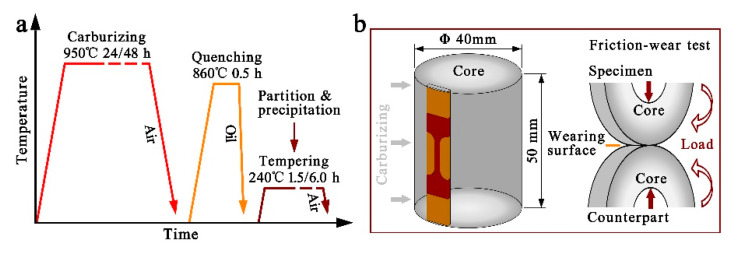
(**a**) is a schematic diagram of the carburizing–quenching–tempering treatment; (**b**) shows the specimen for carburization with the sampling position for tensile test and a sketch of friction-wear test for evaluating wear resistance.

The investigated 18Cr2Ni4WA steel was prepared by vacuum induced melting under Ar atmosphere with the composition Fe-0.16C-1.5Cr-4.25Ni-0.27Si-0.45Mn (wt.%). Specimens with a size of Φ 40 mm × L 50 mm were carburized at 950 °C for 24 and 48 h using nitrogen and methanol as carburizers. The carbon potential during the first carburizing stage (12 h) was about 1.2 wt.%. In the second diffusing stage for 12 h, the carbon potential was set as 1.0 wt.%. The specimens were then cooled in air to room temperature. A sketch of the carburizing–quenching–tempering treatment and specimens for carburizing, tensile test and friction-wear test is shown in Figure 1a,b. The as-carburized specimens were then held at 860 °C for 0.5 h and subsequently quenched in oil to room temperature. Finally, the as-quenched specimen carburized for 24 h was tempered at 240 °C for 1.5 h, named C24-Q-T1.5, while tempering for as-quenched specimens carburized for 48 h was carried out at 240 °C for 1.5 h and 6.0 h, named C48-Q-T1.5 and C48-Q-T6.0, respectively.

Wear resistance of the specimens was evaluated by using a ring-on-ring type friction-wear test machine (MMS-2A) (Jingcheng, Jinan, China) under an unlubricated condition by dry rolling friction at room temperature with a load of 1800 N and a rotation rate of 200 rpm. The sizes of the test ring and counterpart ring were designed in accordance with YBT 5345-2006 [33]. The contact regions for friction wear of the test ring and counterpart ring were ensured to be a carburized surface, of which the surfaces were polished with 2000 grit SiC abrasive paper. All specimens were ultrasonically cleaned with ethanol and dried by a blower before and after the test. The total period for the friction-wear test was 1.0 × 10^6^ cycles, and weight loss was measured in intervals of 0.2 × 10^6^ cycles.

The microstructure of the specimens before and after the friction-wear test was characterized by optical microscopy (OM, Axio Imager M2m) (Zeiss, Jena, Germany) and scanning electron microscopy (SEM, SU8220) (HITACHI, Chiyoda, Japan). The OM and SEM specimens were mechanically ground with 400–1500 grit SiC abrasive papers and then vibration polished and was subsequently etched with 4% Nital reagent. The hardness distribution from surface to core in the carburized layer of the specimens was measured by using a Vickers Hardness Tester (THV-1MDT) (Times Jiufeng, Beijing, China) with an applied load of 1.0 kg for 10 s. The tensile test was carried out at room temperature on a universal material testing machine (CMT5105) (MTS, Eden Prairie, MN, USA) with a displacement rate of 1 × 10^−3^ s^−1^ for which specimens with a gauge size of 10 mm × 2 mm × 1 mm were machined out from the depth of 0.5 mm below the surface and then mechanically ground and polished. An extensometer with the gauge length of 10 mm was used during the tensile test.

## 3. Results and Discussion

### 3.1. Microstructure and Mechanical Property

**Figure 2:**
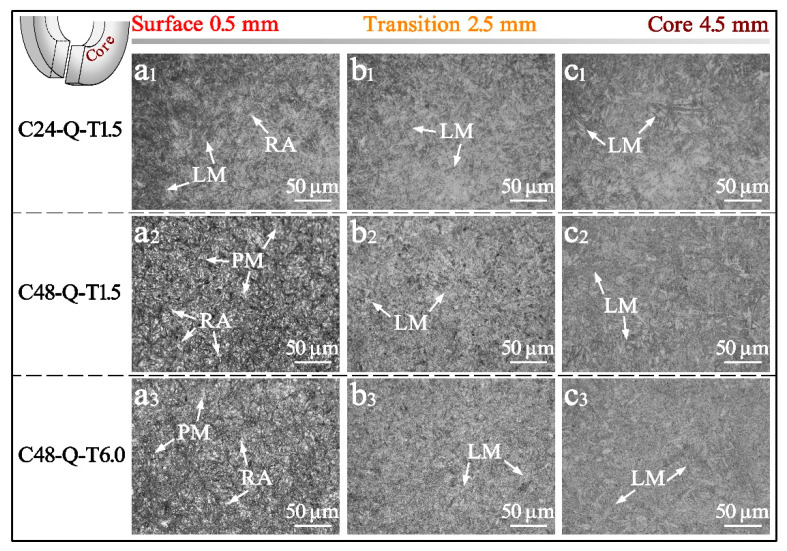
OM images from surface to core in the carburized layer of the specimens processed with different carburizing/tempering durations, where LM, PM and RA represent lath martensite, plate martensite and retained austenite, respectively.

Evident differences could be observed from the surface to the core in the carburized layer of the specimens processed with different carburizing/tempering durations, as shown in Figure 2. A microstructure composed of dense martensite with lath/plate morphology and retained austenite was obtained in the surface, while blocky distributed lath-alike martensite could be observed in the transition and core. Companying the increase in carburizing/tempering duration, the martensite lathes or plates gradually coarsened from surface to core. In comparison to specimen C24-Q-T1.5, long-durations of carburizing with subsequent treatment generated larger areas of coarse carbon partition and retained austenite at the surface of specimens C48-Q-T1.5 and C48-Q-T6.0, with higher carbon contents in the carburized layer, as shown in Figure 2(a1–a3). Moreover, on the basis of micro-scale characterization for precipitates via transmission electron microscopy in previous work [34], larger amounts of carbon precipitation were produced in the surface and transition of specimen C48-Q-T1.5 when compared with specimen C24-Q-T1.5 due to different carburizing durations shown in Figure 2(b1,b2), while specimen C48-Q-T6.0 obtained the largest precipitation, Figure 2(b3), which was due to the long periods of carburizing/tempering. Nevertheless, neither evident differences could be observed in the core of the specimens, as shown in Figure 2(c1–c3), nor in the evolution and transition of tempered martensite, which was supposed to be revealed via mechanical properties, as illustrated in the following section.

**Figure 3:**
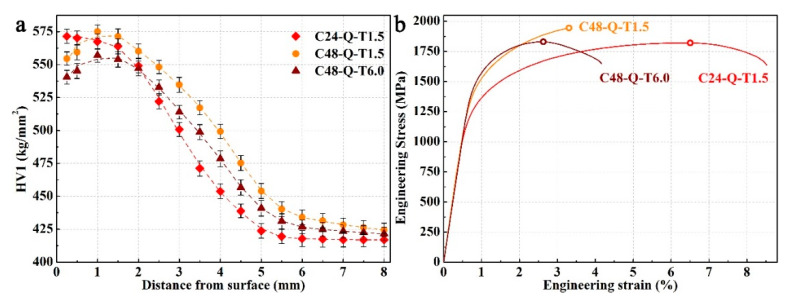
Hardness distribution in the carburized layer of the specimens processed with different carburizing/tempering durations (**a**) and engineering stress–strain curves of the surface layer of the specimens (**b**).

It is worth noting from Figure 3a that the carburized layer achieved a favorable hardened depth of about 2 mm via the carburizing–quenching–tempering process, from which specimen C48-Q-T1.5 acquired the highest hardness while the lowest hardness was obtained by specimen C24-Q-T1.5 due to different carburizing durations. In comparison, long periods of carburizing/tempering produced a medium hardness in specimen C48-Q-T6.0. The highest hardness of 570 HV for a surface lies in specimen C24-Q-T1.5, while the hardness for its core is the lowest at 417 HV. The hardness is 559 HV for the surface and 434 HV for the core of specimen C48-Q-T1.5, which is relativly higher than specimen C48-Q-T6.0 with 545 HV for its surface and 426 HV for its core. It could be deduced that the lower hardness for the surfaces of specimens C48-Q-T1.5 and C48-Q-T6.0 is a result of the relatively high volume fraction of retained austenite and long periods of tempering, while the hardness from the transition to the core gradually reduces with decreasing carburizing time or increasing tempering duration.

Greater differences could be observed between the mechanical properties of the surface layer of the specimens, as shown in Figure 3b and Table 1. Specimen C48-Q-T1.5 achieved the highest elastic modulus and below it is specimen C48-Q-T6.0; the lowest elastic modulus was observed for specimen C24-Q-T1.5. Long periods of carburizing introduced high solid solution carbon in the martensitic matrix, contributing to large obstructions for lattice friction and dislocation migration induced by carbon segregation to a precipitate and the formation of a dislocation loop during deformation, on the basis of the characterization for specimens after mechanical tests in previous studies [34] in which specimens carburized for 48 h acquired high yield strengths. In comparison, long periods of tempering produced high volume fractions of non-deformable precipitates with large sizes in specimen C48-Q-T6.0 [34], resulting in larger resistances for dislocation migration and plastic deformation and giving rise to its highest yield strength; on the other hand, specimen C24-Q-T1.5 achieved the lowest yield strength. A similar ultimate tensile strength was obtained by specimens C24-Q-T1.5 and C48-Q-T6.0, which could be attributed to the shorter durations of carburizing for the former and the long periods of tempering for the latter.

In addition, deformable precipitates with small sizes were produced in specimen C48-Q-T1.5 during short-period tempering [34], enabling dislocations relative to shear and migrates due to which limited strain hardening and plastic deformation could be obtained until the threshold stress for brittle fractures. Hence, uniform elongation and elongation to fracture, i.e., plasticity of specimen C48-Q-T1.5, are reversely poor with regard to the other two specimens, which is difficult for enduring long periods of heavy-load cyclic rolling contact. Combined with high strength and hardness produced by long-durations of carburizing and favorable plasticity and toughness generated during long-durations of tempering, specimen C48-Q-T6.0 obtained favorable comprehensive mechanical properties and may be well qualified with eminent wear resistance. Therefore, different mechanical properties could be generated in the investigated steel through carburizing–quenching–tempering processes with different carburizing/tempering durations, which may have prominent influences on wear resistance, as discussed in the following section.

### 3.2. Wear Resistance Analysis

**Figure 4:**
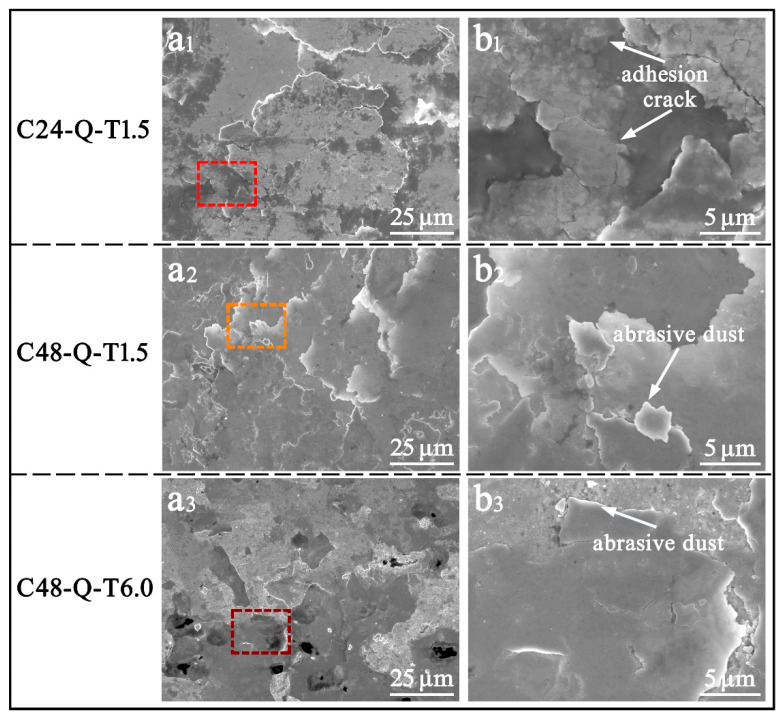
SEM images of the worn surface of the specimens processed with different carburizing/tempering durations; (**b1**–**b3**) is the magnified map of the selected region in (**a1**–**a3**).

The worn contact surface morphology of the specimens processed with different carburizing/tempering durations was characterized as shown in Figure 4. The severest wear was endured by specimen C24-Q-T1.5 compared with the other two specimens. Bulk flaking could be observed on its worn surface, shown in Figure 4(a1), and adhesion debris and cracks propagated from subsurface can be found, as shown in Figure 4(b1). The carburized layer with low solid solutions of carbon lacked strength, hardness and the ability of strain hardening, and its effects of carbon pinning on dislocation and the intrinsic strength of martensite were also poor; this rendered it difficult for specimen C24-Q-T1.5 to endure severe strain and deformation. Thus, the initiation, interaction and propagation of cracks in the subsurface were easily induced, and wear spalling and flaking would be generated after long periods of heavy-load cyclic rolling contact. Conversely, high yield strength and tensile strength were introduced on the carburized layer of specimen C48-Q-T1.5; due to this, a few abrasive flakes or dusts could be found on its worn surface, as shown in Figure 4(a2,b2). High solid solution carbon gave rise to severe pinning effects on dislocation migration, while deformable precipitates with small sizes emerged from the matrix during the short period of tempering and enabled dislocations to shear and migrate under high contact loads [34]. Thereby, accompanying dislocation density and strain hardening when approaching saturation after long periods of heavy-load cyclic rolling contact, a sacrifice of plasticity and toughness was induced, and brittle spalling emerged. In comparison, favorable comprehensive mechanical properties were obtained by specimen C48-Q-T6.0 processed with long durations of carburizing/tempering. Only a few abrasive dusts could be observed on its worn surface, while almost no flaking or pilling could be found, as shown in Figure 4(a3,b3). Carbon partitions in the martensitic matrix generated non-deformable precipitates with large sizes during long periods of tempering, which compelled dislocations to bypass precipitates, giving rise to the formation of dislocation loop and high resistance for migration as well as limited strain hardening [34]. Deformation energy was continuously consumed during long periods of heavy-load cyclic rolling contact. Hence, favorable wear resistance was obtained by specimen C48-Q-T6.0.

The investigation of the formation of rolling contact fatigue could be summarized in several stages [35,36,37,38] in terms of microstructure evolution under long periods of high cyclic contact stress: In the first stage, the rough contact surface is severely worn and gradually achieves a steady interaction due to abrupt increases in stress. In the second stage, microstructure deformations and micro-cracks are initiated due to the accumulation of strain below contact surfaces during a definite period of steady friction wear. In the third stage, the fatigue cracks interact, and a large crack is induced and gradually propagates to the surface, resulting in stress concentration areas and aggravation of wear. Finally, abrasive spalling or flaking is formed after a long period of wear. Therefore, morphologies of subsurface cracks are prominent to wear resistance.

**Figure 5:**
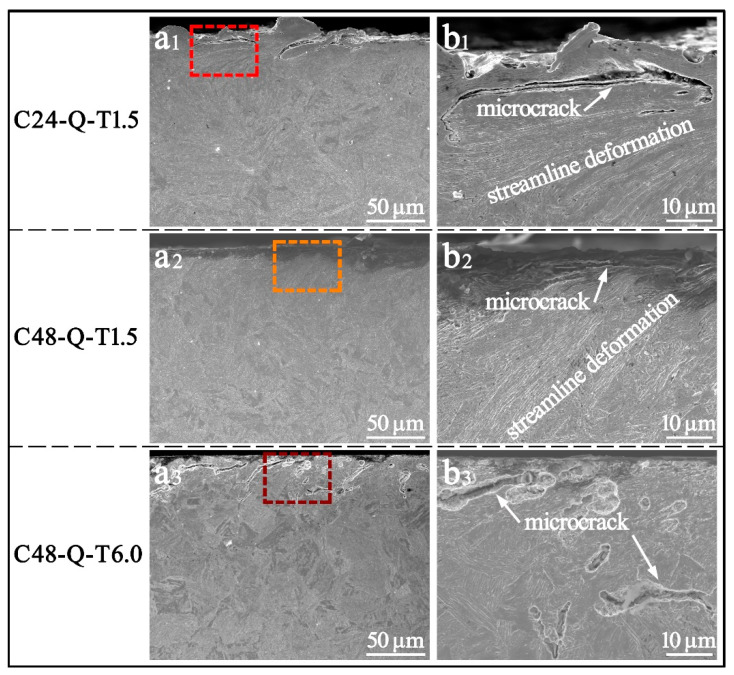
SEM images of the worn section of the specimens processed with different carburizing/tempering durations; (**b1**–**b3**) is the magnified map of the selected region in (**a1**–**a3**).

The microstructural features in the circumferential section of the worn layer of the specimens processed with different carburizing/tempering durations were characterized, as shown in Figure 5. Severe wear and destruction of surface integrity could be observed on the worn surface of specimen C24-Q-T1.5 due to low hardness, as shown in Figure 5(a1,b1), and quite a few micro-cracks as well as a large fatigue crack could be found in the subsurface, mainly attributed to its poor strength for enduring strain and deformation. In contrast, little wear could be found on the worn surface of specimens C48-Q-T1.5 and C48-Q-T6.0 processed with long durations of carburizing, as shown in Figure 5(a2,a3). High solid solution carbon generated severe pinning effects on dislocation migration, enabling the specimens with high strength and hardness to endure friction wear. However, an evident difference could be observed between the depth of cracks in the subsurface of specimens C48-Q-T1.5 and C48-Q-T6.0, as shown in Figure 5(b2,b3). It is obvious that the crack below the surface of the former propagated faster compared to the latter, implying that specimen C48-Q-T1.5 has a higher rate of wear and is more likely to form spalling or flaking before the other due to its high strength and hardness but low toughness originating from short-period tempering. A favorable wear resistance was achieved by specimen C48-Q-T6.0, which was carburized and tempered for long durations. It was enabled with better abilities with respect to strain hardening as well as good plasticity and toughness. The non-deformable precipitates with large sizes compelled dislocations to bypass and migrate, creating large obstructions for dislocation migration and plastic deformation under long periods of heavy-load cyclic rolling contact and consuming deformation energy, due to which the formation, interaction and propagation of fatigue cracks and wear pitting/spalling were impeded.

**Figure 6:**
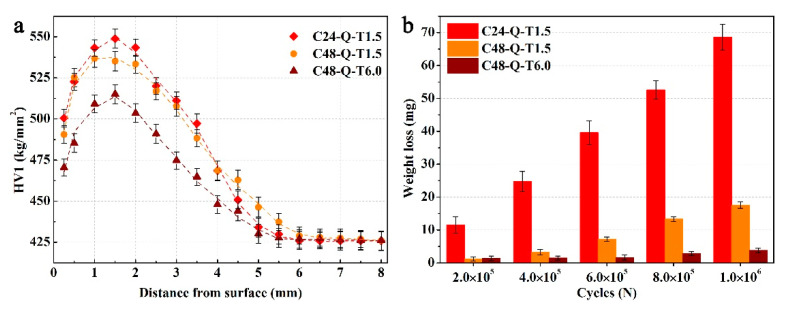
Hardness distribution from surface to core of the worn layer (**a**) and weight loss (**b**) of the specimens processed with different carburizing/tempering durations after friction-wear test.

A gradient decrease in hardness is shown in Figure 6a along the depth below which the surfaces of the specimens were worn after the friction-wear test. The destruction of surface integrity resulted in the softening of the surface layer, while hardness for the core obtained a slight augment mainly due to accumulation of microstrains in the center. In addition, it was observed that the hardness for specimens processed at longer durations of carburizing decreased more compared to specimen C24-Q-T1.5, which could be attributed to the decrease in carbon pinning effects on dislocation migration and reduction in the intrinsic strength of martensite due to the segregation of carbon along the microstructural interface and formation of tempered martensite during cyclic rolling contact.

The weight loss as a function of cyclic cycles for the specimens during the friction-wear test was measured, as shown in Figure 6b. Specimen C24-Q-T1.5 endured the highest wear due to the low carbon content in the carburized layer, with poor abilities of strain hardening. In comparison, specimen C48-Q-T1.5 was competent in enduring friction wear during initial periods of friction wear but was not capable of assimilating accumulated strain caused by long periods of heavy-load cyclic rolling contact due to its high strength and hardness but poor plasticity. Hence, the initiation of fatigue crack and brittle flaking was untimely compared with specimen C48-Q-T6.0, which presented the lowest weight loss. Apart from the high strength and hardness produced on the carburized layer during long durations of carburizing, non-deformable precipitates with large sizes generated during longer durations of tempering created larger resistances for dislocation migration and plastic deformation [34], which continuously consumed deformation energy and impeded the formation, interaction and propagation of fatigue cracks and wear pitting/spalling. That is, favorable comprehensive mechanical properties and wear resistance were obtained by specimen C48-Q-T6.0.

### 3.3. Fatigue-Wear Model

Apart from experimental investigation, theoretical analysis is presumed for the strengthening–toughening process in order to optimize comprehensive mechanical properties as well as wear resistance of heavy-duty gear. As far back as 1963, a dislocation based model for plastic yield and fracture under contact load was proposed by Bily, Cottrell and Swinden [39] on the basis of which micro-cracks may well emerge as the formation of dislocations piling up around the stress concentration area. Along with similar dislocation based models raised later [40], experimental studies were further carried out, which validated that micro-cracks were prone be to initiated and propagated from hard particles with dislocation pile ups below the contact surface. Moreover, in terms of elastic-plastic mechanics theory, the dislocation based model was improved and implemented for analyzing microstructure evolution and wear features in the subsurface under cyclic contact fatigue-wear conditions [41]. Recently, Huang et al. [42] explored the formation of rolling contact fatigue cracks and cyclic line contact stresses under dry conditions and provided a schematic of fatigue damage evolutions on the basis of the dislocation based model [39,40,41] and experimentally characterized wear features. The potential damage mechanism and causes for differences in crack propagation were finely revealed.

**Figure 7:**
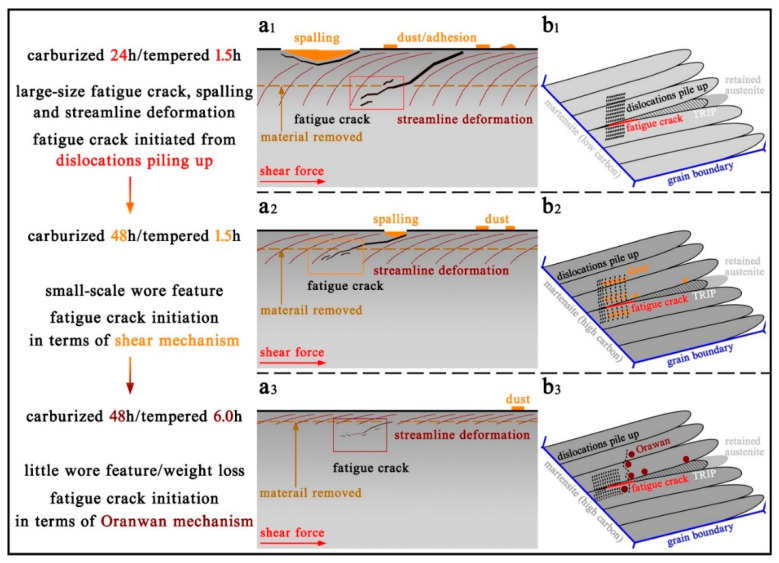
Fatigue-wear model corresponding to the specimens processed with different carburizing/tempering durations; (**b1**–**b3**) is the magnified map of the selected region in (**a1**–**a3**).

In this study, on the account of wear features and wear resistance analyzed in the former sections, a fatigue-wear model is proposed, shown in Figure 7, on the basis of the dislocation based model [39,40,41]. A microstructure composed of martensite, retained austenite and precipitate could be prepared by carburizing–quenching–tempering processes, while different solid solution carbon is introduced during disparate carburizing durations, and diverse precipitates and retained austenite could be generated with different tempering durations. Favorable comprehensive mechanical properties and wear resistance are presumed to endure long periods of heavy-load cyclic rolling contact.

During the initial period, severe wear is generated on rough surfaces with increasing cyclic contact stress; subsequently, steady interaction between the worn surfaces proceeds for a definite duration, during which dislocation migration, strain hardening and plastic deformation continuously accumulate, giving rise to the formation of micro-cracks around the microstructural interface and stress concentration areas. Large-size spalling and fatigue crack, large-area streamline deformations and dense dust and adhesion would emerge after long periods of heavy-load cyclic rolling contact in the specimen C24-Q-T1.5, as shown in Figure 7(a1). Figure 7(b1) shows that the formation of fatigue cracks mainly originated from dislocations piling up along the martensitic interface with low carbon content processed with short durations of carburizing/tempering. The martensitic microstructure with high solid solution carbon produced by long durations of carburizing generated severe carbon pinning effects on dislocation and strain hardening, inducing high strength and hardness with respect to resisting wear. By accompanying friction-wear, cumulative dislocations generated around the martensitic lathes/plates interfaces and shear-deformable precipitate with small sizes, due to which strain hardening approaches saturation while extra deformation energies produced by continual cyclic rolling contact could not be consumed, contribute to the initiation, interaction and gradual propagation of fatigue cracks from stress concentration areas in the subsurface, where dense dislocation piles up. As shown in Figure 7(a2), wear spalling and subsurface cracks are much smaller, and little streamline deformations and dust could emerge in specimen C48-Q-T1.5, and the origin of fatigue cracks is mainly attributed to stress concentration areas of deformable precipitates with small sizes and dislocations piling up, shown in Figure 7(b2). Non-deformable precipitates with large sizes are obtained with a slight sacrifice of strength, but an optimization of toughness induced by carbon partitioning during long durations of tempering following long periods of carburizing [34], due to which dislocations are compelled to bypass precipitates, results in large resistances for dislocation migration and plastic deformation. Accompanying the accumulation of dislocation density and micro-strains approaching saturation, the formation, interaction and propagation of fatigue cracks emerged. However, deformation energy could be gradually consumed due to the large obstructions of cumulative strain induced by large precipitates in which wear pitting and wear spalling were impeded. Hence, favorable comprehensive mechanical properties and wear resistance were obtained by specimen C48-Q-T6.0; thus, little wear features would be generated, as shown in Figure 7(a3,b3) demonstrates that the pinning effect of non-deformable precipitate with large sizes and the dislocations piling up dominantly gave rise to the formation of fatigue cracking in the martensitic matrix.

## 4. Conclusions

In this paper, the microstructure, mechanical properties and wear resistance of carburized–quenched–tempered steel processed with different carburizing/tempering durations before and after friction-wear tests have been studied, and its fatigue-wear mechanisms were analyzed. The following conclusions were drawn.

A microstructure composed of lath/plate martensite, retained austenite and precipitate was obtained on the surface of the investigated steel prepared by the carburizing–quenching–tempering process, while lath martensite and retained austenite were produced in the core. A favorable hardened carburized layer with a depth of about 2 mm and corresponding microstructure/hardness distribution could be due obtained via the aforementioned carburizing–quenching–tempering process. In comparison, specimen C48-Q-T6.0 endured the lowest weight loss and slightest wear features during friction wear. Long periods of carburization for 48 h with subsequent quenching processes produced a high-carbon content matrix with high dislocation density and strength, and the following long-duration tempering for 6.0 h generated favorable toughness and introduced non-deformable precipitates with large sizes due to which deformation energies were continuously consumed, and crack propagation and macro-strain were impeded during heavy-load cyclic rolling contact. Therefore, favorable comprehensive mechanical properties and wear resistance were obtained by the specimen processed with long durations of carburizing/tempering.

A fatigue-wear model was proposed to describe fatigue crack evolution and fatigue-wear mechanism on the basis of wear features. Severe wear was generated on rough surfaces from the initial friction wear, and steady interaction between the worn surfaces proceeded for a definite duration. Accompanying the increase in stacking dislocation, strain hardening and micro-strain continuously accumulated. Thereby, micro-cracks emerged around the microstructural interface and stress concentration areas. Deformable precipitates with small sizes enabled dislocations to shear and migrate with low resistance, while non-deformable precipitates with large sizes created large resistances for dislocation migration and plastic deformation. Deformation energy was continuously consumed during long periods of heavy-load cyclic rolling contact. The formation, interaction and propagation of fatigue crack and wear pitting/spalling were impeded.

## Figures and Tables

**Table 1:** Mechanical Properties of the Surface Layer of the Specimens.

Process	E (GPa) ^1^	σ0.2% (MPa) ^2^	σb (MPa) ^3^	δu (%) ^4^	δf (%) ^5^
C24-Q-T1.5	204.7	1286.9	1820.8	6.34	8.52
C48-Q-T1.5	211.4	1467.4	1945.1	3.30	3.30
C48-Q-T6.0	206.8	1542.8	1829.9	2.61	4.16

^1^ Elastic modulus; ^2^ yield strength; ^3^ ultimate tensile strength; ^4^ uniform elongation; ^5^ elongation to fracture.

## Data Availability

The data presented in this study are available on request from the corresponding author.
